# Evolving Appetites: Current Evidence and Future Perspectives in Terms of Meat Substitutes in Europe

**DOI:** 10.1002/fsn3.4753

**Published:** 2025-01-12

**Authors:** Muhammad Adzran Che Mustapa, Yasmina Baba, Smita Dash Baishakhy, Zein Kallas

**Affiliations:** ^1^ Centre for Agro‐Food Economics and Development‐UPC‐IRTA (CREDA) Universitat Politècnica de Catalunya (UPC) Castelldefels Spain; ^2^ Organització i Recursos Humans Universitat de Barcelona Barcelona Spain; ^3^ Department of Agricultural Extension Education Sylhet Agricultural University Sylhet Bangladesh

**Keywords:** alternative protein source, consumer behavior, Europe, meat substitutes, novel protein foods

## Abstract

Consumers are increasingly aware of the environmental and health impacts of their food choices, leading to changes in consumption behavior. This study examines the consumption patterns and behaviors of European consumers regarding meat substitutes and identifies factors influencing their acceptance as alternative protein sources. The study involved 5000 participants from four European countries—France, Germany, Italy, and Spain with data extracted from the Mintel consumer database in 2024. Results indicate that over 50% of consumers in these countries have reduced their intake of red meat, with a notable portion of German consumers adhering to a vegan diet. Across the sample, females significantly differ from males, as they consume less meat, tend to limit their meat intake, and show a greater interest in reducing meat consumption. Baby Boomers predominantly consume more meat and show less interest in reducing their meat intake compared to younger generations. Economic and sustainability aspects are key perceptions driving the perceived benefits of reducing meat consumption. Additionally, preferences for locally sourced products, meat‐like taste and texture, and natural ingredients are primary considerations when purchasing meat substitutes. Fish substitutes were rated as popular meat substitutes in France, Italy, and Spain, while breaded or battered meat/poultry substitutes were the most popular choice in Germany. Insights from this study are valuable for market researchers and the food industry, offering guidance on supplying appealing and sustainable protein alternatives that encourage a shift toward healthier and more sustainable consumption behavior.

## Graphical abstract

This article analyzes European consumers' pattern and behavior of meat substitutes, revealing a significant shift in dietary habits driven by environmental and health concerns. Findings from 5000 participants across France, Germany, Italy, and Spain highlight motivations such as sustainability and natural ingredients, influencing preferences for plant‐based protein alternatives. The study underscores opportunities for food industries to cater to evolving consumer preferences toward sustainable and healthier eating choices.

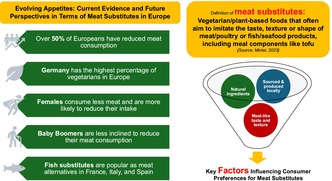

## 1 Introduction

The intense industrialization of the agricultural system and the globalization of the food market over the last decade have led to a rapid expansion in industrial meat production systems (Weis 2013). Factors such as affordability and mass availability have also encouraged consumers to incorporate increasing volumes of meat into their diets leading to higher meat consumption (Siegrist, Michel, and Hartmann 2024). However, numerous studies have consistently linked high meat consumption, particularly red and processed meat, to an increased risk of various diseases (Bellavia, Stilling, and Wolk 2016; Demeyer et al. 2016; Larsson and Orsini 2013; Li et al. 2024). According to Murray, Aravkin, and Zheng (2020), the Global Burden of Diseases, Injuries, and Risk Factors Study (GBD) 2019 found that eating unprocessed red meat was linked to 896,000 deaths and 23.9 million disability‐adjusted life years worldwide in 2019. Furthermore, studies have linked meat production and consumption to negative environmental impacts, ethical issues, and compromised animal welfare (Graça, Calheiros, and Oliveira 2014; Steinfeld 2006). In addition, reducing meat consumption is crucial because of the unsustainable increase in demand for dairy and meat over the long term (de Bakker and Dagevos 2012). Additionally, in current times, the fear of animal‐borne diseases and public concerns about animal suffering in meat production have led consumers to express an interest in decreasing their intake of animal products (Fonseca and Sanchez‐Sabate 2022). Consequently, Matos et al. (2022) stressed utilizing innovation and technology for developing new approaches to effectively tackle the impact of the meat production system on our environment under the global threat of climate change. Under this context, leading institutions such as the World Health Organization (WHO), the World Cancer Research Fund (WCRF), the EAT‐Lancet Commission, and the US Departments of Health and Human Services and Agriculture have been recommending limiting red meat intake in our day‐to‐day diet for health reasons, sustainability, and ethical considerations (Lescinsky et al. 2022).

Several strategies have been proposed to improve sustainability in the meat industry and the most viable options are reducing meat consumption, developing sustainable alternative protein sources and adopting plant‐based/vegan meat alternatives (Tyndall et al. 2024). To this date, numerous studies have come in support of adopting alternative protein sources as the efficient and acceptable approach in reducing meat consumption and to address challenges related to both public health and environmental sustainability (Graça, Godinho, and Truninger 2019; Hartmann and Siegrist 2017; Macdiarmid, Douglas, and Campbell 2016). Several studies also have explored the potential of alternative proteins and meat substitutes in the context of environmental sustainability and health, highlighting benefits such as reduced environmental impact and improved health outcomes (Bryant 2022; Springmann et al. 2018). To provide a context, the global market for meat alternatives was worth USD 10.1 billion in 2023 and is forecast to exceed USD 233.87 billion by 2030, with a steady 42.1% compound annual growth rate (CAGR) during that period (Market Analysis Report 2024). It is also predicted that by 2035, one in ten servings of dairy, seafood, meat, and eggs globally will contain alternative proteins (BCG 2021). These findings highlight the potential to promote alternative proteins and meat alternatives in reducing our carbon footprint and in supporting sustainable efforts for our health and environment.

**FIGURE 1:**
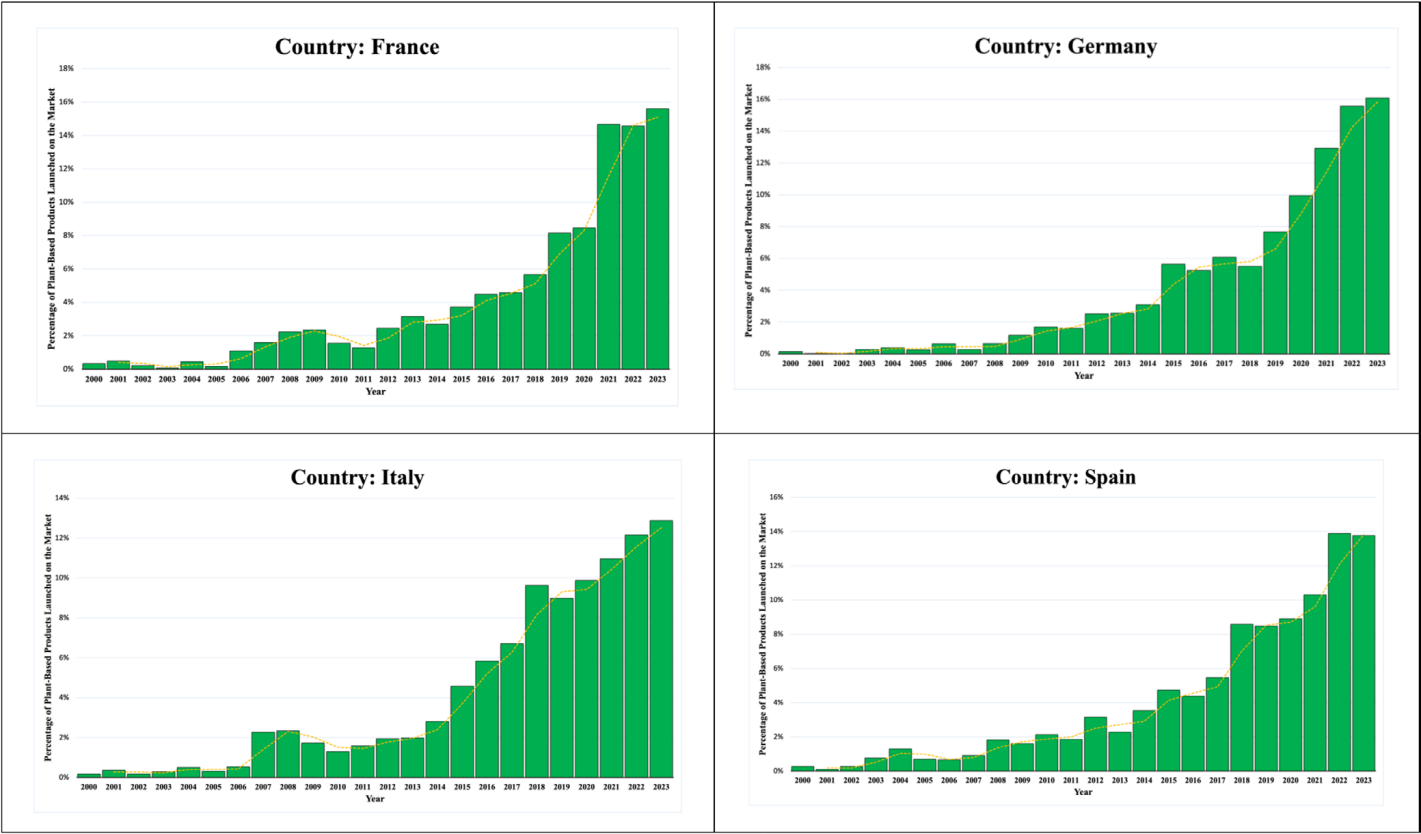
Trends in the acceptability of plant‐based products launched in European markets (*Source:* Mintel GNPD Data). The *x*‐axis represents the years from 2000 to 2023, while the *y*‐axis indicates the percentage of plant‐based products launched in each country during the respective years.

In current times, consumer awareness of alternative proteins and meat substitutes is rapidly increasing, as more and more individuals are acknowledging the health and environmental benefits of such alternative‐protein sources (Hartmann and Siegrist 2017). However, meat consumption is still considerably high coupled with a larger volume of industrialized production (Cai et al. 2024). Besides, since consumers tend to be reluctant to welcome drastic fundamental changes in their diet, the introduction of meat alternatives and increasing production of these alternatives alone are not sufficient to reduce meat consumption (Hartmann and Siegrist 2020; Tarrega et al. 2020). Therefore, to promote sustainable food consumption behavior among consumers, it is crucial to understand consumers' motives and the determining factors that influence their acceptance of such alternative protein sources (Hartmann and Siegrist 2017; Siegrist, Michel, and Hartmann 2024). A recent survey by the Good Food Institute Europe GFI (2022) reported that the alternative proteins and plant‐based food industry particularly in Europe has experienced tremendous growth in recent years and is becoming popular. The industry is currently valued at €5.8 billion, with both total sales value and unit sales increasing by 21% between 2020 and 2022 (GFI Europe 2022). In addition, Figure 1 illustrates the availability of plant‐based products launched in the market across four European countries—France, Germany, Italy, and Spain—based on data extracted from the Mintel Global New Products Database (GNPD) for the period 2000 to 2023. As meat substitutes offer a sustainable food consumption option, and cover a significant portion of European consumers diet, evaluating European consumers consumption behavior and attitude toward meat‐alternatives are crucial to facilitate mass‐acceptance of such sustainable and healthier meat alternatives at consumer label.

There are some studies on meat substitutes and consumer behavior (Kopplin and Rausch 2022; Li et al. 2023; Tosun et al. 2021), particularly regarding the consumption pattern of European consumers (Andersson and Hannah 2023; Hartmann and Siegrist 2017; Henn et al. 2022) that provided valuable insights and existing hinders. For example, Hoek et al. (2011) identified key barriers such as cost and taste preferences, as well as drivers such as health concerns, influencing meat substitute consumption in the UK and the Netherlands. Additionally, Lemken, Spiller, and Schulze‐Ehlers (2019) explored consumer acceptance of meat substitutes being replaced by legume products as a sustainable food consumption option in two countries: New Zealand and Germany. Besides, Michel, Hartmann, and Siegrist (2021) discuss consumers' associations, perceptions, and acceptance of meat and plant‐based meat alternatives on the part of German consumers. However, despite the rising popularity of meat substitutes and alternative proteins in European countries which, to the best of our knowledge, is expected to continue to grow, there has been limited research on aspects of consumer consumption behavior focusing on their acceptance of alternative proteins or meat substitution products in Europe. But, considering the increasing demand for meat alternatives in Europe, there is a clear need for in‐depth evaluation on European consumers consumption patterns, attitude and acceptance of such alternative proteins. Therefore, the purpose of this study was to evaluate European consumers' consumption patterns and their willingness to reduce meat consumption in the future. To achieve this aim, the main objectives were to investigate the current consumption pattern of European consumers in terms of their dietary preferences and their attitudes toward meat (red meat, poultry) and meat substitutes consumption. Subsequently, the study examined their underlying motivations in making dietary decisions and the purchasing behavior related to meat substitutes. The final objective was to explore the factors influencing the selection of meat substitutes (if consumed). Findings derived from this study will help to identify the most promising alternative proteins and meat substitutions with the most potential as an efficient strategy for changing European consumers' food behavior toward sustainable and healthier directions. Besides, it also contributes to the existing literature by providing comprehensive evidence on the current state of meat alternatives across the European food market and cross‐country differences in consumer demand and acceptance of these alternatives food choices.

The outline of the rest of the paper is as follows: In Section 2, we present the research method used in the study. Section 3 presents a detailed analysis of the empirical results, including statistical and comparative analyses across sociodemographic variables. Finally, Section 4 presents a discussion of the findings, implications, limitations, and future directions derived from this study.

## 2 Materials and Methods

### 2.1 Data Source and Data Collection

Consumer data for this work was extracted from Mintel's GNPD in January 2024. Mintel is an international market research company that collects information on product characteristics of newly introduced products in supermarkets in various countries and has been curating a large food database since 1972. We chose to use this database as our source of consumer data because it is an innovation‐oriented database providing detailed information on the available food product market supply and consumers' acceptance of the new products. With other databases, it is not possible to evaluate how market supply for new innovative products is changing and whether those food innovations are successful for consumers (Ramos‐Souza et al. 2023). Most importantly, the Mintel database has been widely used in similar studies, including those by Petersen and Hirsch (2023) and Petersen, Hartmann, and Hirsch (2021), which focused on meat consumption and alternatives.

In this study, the panelists were not directly recruited from the Mintel GNPD “Meat Substitutes in Europe in 2023” database. Instead, they were sourced through an external consumer panel provider, following a similar approach to Mintel's use of Kantar Profiles for consumer surveys. This provider ensured a representative sample from France, Germany, Italy, and Spain through a quota‐sampling approach. Quotas were based on key demographic variables such as age, gender, and region to reflect the national demographics of each country. Participants were randomly selected within these quotas to maintain representativeness across demographic groups. The final sample sizes were as follows: France (*n* = 1000), Germany (*n* = 2000), Italy (*n* = 1000), and Spain (*n* = 1000), enabling a comprehensive analysis of consumer behavior in each country.

For this study, meat substitutes and dishes made using meat substitutes were defined as: “Vegetarian/plant‐based foods that often aim to mimic the taste, texture, or appearance of meat, poultry, or fish/seafood products, including meal components such as tofu.” This definition aligns with substantial research that considers tofu a meat substitute in the European context. For instance, Petersen and Hirsch (2023) conducted a comparative study on the nutritional composition of red meat, poultry meat, and emerging meat substitutes across five major European countries—France, Germany, the UK, Italy, and Spain. In their analysis, tofu was classified as a vegan meat substitute as it fulfills the functional role of meat in meals. Similarly, Tonheim et al. (2022) assessed the macronutrient and salt content of meat and dairy substitutes available in the Norwegian market, categorizing tofu as a plant‐based meat substitute. Hartmann, Furtwaengler, and Siegrist (2022) surveyed Swiss‐German participants to explore consumer perceptions of protein‐rich foods regarding environmental friendliness, health benefits, and naturalness, identifying tofu as a prominent meat substitute. Michel, Hartmann, and Siegrist (2021), in a study involving German participants, also found that terms like “tofu,” “vegan,” and “vegetarian” were strongly associated with meat alternatives, reinforcing tofu's role as a common meat substitute in European markets.

Additionally, consumer samples in this study were segmented based on socio‐demographic profiles, including gender, employment status, income, and generation. All data were entered into the working database for further analysis.

### 2.2 Survey Instruments

The survey questions used in this study were made up of seven questions that were developed and modified based on earlier works (Binnie et al. 2014; Guenther et al. 2005; McAfee et al. 2010; Zur and Klöckner 2014).

First, consumers were asked three sequential questions about their meat consumption behavior. In Question 1, respondents were asked if they eat red meat (such as beef, pork, lamb, or game) or poultry. Only those who answered “Yes” proceeded to the next question, which inquired whether they had limited or reduced their red meat or poultry intake over the past 6 months (Question 2). If respondents answered “No” to this second question, they were then asked if they would be interested in limiting or reducing their red meat or poultry consumption in the future (Question 3). Each of these questions required a yes‐or‐no response.

In Question 4, to examine consumers' motivations for reducing meat consumption, all respondents were presented with seven specific statements (motives) regarding the perceived benefits of reduced meat consumption (Kemper 2020; Lentz et al. 2018; Zur and Klöckner 2014). The survey used a check‐all‐that‐apply (CATA) format, allowing respondents to select all statements they felt were relevant to them.

In Question 5, consumers were asked whether they had purchased any meat substitutes in the previous 6 months (Hoek et al. 2011), with three response options: Yes, No, and Don't know. Question 6 focused on consumers' preferences regarding the types of meat substitutes they consumed. Question 7 examined the factors influencing their decision to purchase meat substitutes, allowing respondents to select up to three options. Only those who answered “Yes” to Question 5 were included in the analysis for Question 7. The questionnaires were presented in the native language of each respective country.

### 2.3 Data Analysis

Descriptive analysis was performed to examine the consumption patterns of meat alternatives and the proportion of consumers from each country. Additionally, consumers' preferences and their heterogeneity in reducing meat consumption, along with their motivations and interest in consuming meat alternatives, were analyzed and presented as percentage shares across different consumer groups.

Significant differences in percentage share values among panelists' attributes were identified using the chi‐square test of independence (Tables 1, 2, 3, 4, 5). This statistical test is commonly used to compare categorical variables and assess whether the observed differences between groups are statistically significant, providing valuable insights into the relationships between attributes.

**TABLE 1:** Meat consumers' proportion and the effect of their socio‐demographic profile (asked of internet users aged 16+).

Do you eat red meat (e.g., beef, pork, lamb, game) or poultry?	France (*n* = 1000)		Germany (*n* = 2000)		Italy (*n* = 1000)		Spain (*n* = 1000)	
All	Yes = 899 (90%)^y^		Yes = 1653 (83%)^x^		Yes = 900 (90%)^y^		Yes = 919 (92%)^y^	
	No/Vegan = 101 (10%)		No/Vegan = 347 (17%)		No/Vegan = 100 (10%)		No/Vegan = 81 (8%)	
Gender	*n*	%	*n*	%	*n*	%	*n*	%
Male	477	92%^a^	978	87%^a^	487	93%^a^	489	92%
Female	521	88%^b^	1012	79%^b^	512	88%^b^	511	92%
Others	2	—	10	—	1	—	0	—
Employment								
Full‐time	447	88%^a^	834	83%	425	89%	474	92%
Part‐time	110	84%^a^	328	83%	169	89%	126	94%
Not working	443	93%^b^	838	82%	406	91%	400	92%
Net monthly household income								
Less than €1500	334	87%^a^	641	83%	358	87%^a^	428	93%
€1500–€2999	315	89%^a^	703	83%	320	93%^b^	344	92%
€3000 or more	233	95%^b^	456	83%	156	94%^b^	135	92%
Prefer not to say/Don't know	118	92%^ab^	200	76%	166	87%^ab^	93	87%
Generation								
Generation Z (25 and under)	160	88%^a^	269	74%^a^	142	88%	130	94%^ab^
Millennials (26–41)	243	87%^a^	507	81%^a^	246	90%	256	94%^a^
Younger Millennials (26–32)	109	85%^a^	217	82%^ab^	95*	89%	106	93%^ab^
Older Millennials (33–41)	134	89%^ab^	290	81%^a^	151	90%	150	95%^ab^
Generation X (42–57)	268	90%^ab^	547	84%^ab^	321	90%	327	91%^ab^
Baby Boomers (58–76)	313	93%^b^	627	87%^b^	274	91%	268	89%^b^
Swing Generation/World War II (77+)	16*	—	50*	—	17*	—	19*	—

*Note:* Data percentages are not displayed for groups with fewer than 75 respondents, as the sample size is too low to provide meaningful insights and is represented as (−). Proportion Representation: Percentages reported in the tables are calculated within each socioeconomic group and represent the distribution of responses within these groups, rather than the overall percentage of the total population answering the question. For superscript: a, b: Different letters in the same column indicate statistically significant differences (*p* < 0.05); ab: Indicates no significant difference from both “a” and “b” values. Meanwhile, for superscript: x, y: Different letters in the same row indicate statistically significant differences (*p* < 0.05); xy: indicates no significant differences from both “x” and “y” values.

* Small sub‐sample: 75 to 100 is a low base size.

For Tables 6 and 7, no statistical tests for significant differences were conducted, as these tables reflect consumer selections based on predefined answer options. All statistical analyses were carried out using SPSS software, version 29.

## 3 Results

### 3.1 Meat Consumers' Proportion and the Effect of Their Socio‐Demographic Profile

Table 1 presents the findings on the meat consumption behavior of European consumers and the impact of their socio‐demographic profiles in France, Germany, Italy, and Spain. The results indicate a widespread preference for red meat consumption across all surveyed countries, with Spain having the highest proportion of meat consumers (92%), followed by France (90%), Italy (90%), and Germany (83%) (Table 1). Interestingly, Germany reported the largest percentage of respondents identifying as vegans (17%), characterized by the avoidance of meat, fish, and all animal products, including dairy and eggs (Table 1).

A country‐wide comparison revealed that Germany stands out, with the proportion of meat consumers being statistically significantly lower than in France, Italy, and Spain (Table 1). Regarding gender differences, males consistently exhibited higher levels of red meat consumption than females in France, Germany, and Italy. However, in Spain, the gender‐based differences in meat consumption were not statistically significant (Table 1).

The relationship between employment status and meat consumption varied across countries. In France, non‐working individuals, such as retirees, were more likely to consume meat compared to those employed full‐time or part‐time. This may be attributed to retirees having stable financial resources, such as pensions, that enable them to maintain their dietary habits (Table 1). In contrast, this trend was less evident in Germany, Italy, and Spain, where meat consumption did not significantly differ across employment categories.

Household income levels demonstrated a strong influence on meat consumption. Higher‐income households consistently consumed more meat, with the most pronounced differences observed in France and Italy (Table 1). This suggests that greater disposable income facilitates higher meat consumption. While this finding might seem contradictory to the higher consumption among non‐working individuals, it highlights two distinct factors influencing meat consumption: stable financial support among retirees and increased purchasing power in higher‐income households.

Generational differences also played a notable role in shaping meat consumption patterns. Baby Boomers exhibited significantly higher meat consumption than younger generations, such as Generation Z, Millennials, and Younger Millennials (Table 1). This trend was particularly prominent in France, Germany, and Spain, suggesting that cultural and generational factors influence dietary habits. Younger generations appear to be shifting toward reduced meat consumption compared to their older counterparts.

### 3.2 Limiting Meat Consumption: Consumers' Preference and Heterogeneity

The survey results on whether consumers had limited or reduced their consumption of red meat and poultry over the past 6 months, as shown in Table 2, reveal notable trends. Across all countries, more than 50% of respondents reported reducing their intake of red meat. France recorded the highest percentage (58%), followed by Germany (55%), Italy (54%), and Spain (50%) (Table 2). A country‐wide comparison highlights that Spain had a statistically significantly lower proportion of individuals reducing their meat consumption compared to France and Germany (Table 2). In contrast, the differences between France, Germany, and Italy were not statistically significant.

**TABLE 2:** Limiting meat consumption: Consumers' preferences and heterogeneity (asked of internet users aged 16+ who eat red meat/poultry).

Have you limited/reduced the amount of red meat/poultry you have eaten in the last 6 months?^1^	France (*n* = 899)		Germany (*n* = 1653)		Italy (*n* = 900)		Spain (*n* = 919)	
All	Yes = 517 (58%)^y^		Yes = 901 (55%)^y^		Yes = 490 (54%)^xy^		Yes = 462 (50%)^x^	
	No = 382 (42%)		No = 752 (45%)		No = 410 (46%)		No = 457 (50%)	
Gender	*n*	%	*n*	%	*n*	%	*n*	%
Male	439	49%^a^	851	48%^a^	451	50%^a^	448	48%
Female	460	66%^b^	796	61%^b^	448	59%^b^	471	53%
Others	0	—	6	—	1	—	0	—
Employment								
Full‐time	395	57%	694	53%	380	54%	435	49%
Part‐time	92*	53%	272	57%	150	50%	118	53%
Not working	412	59%	687	55%	370	56%	366	51%
Net monthly household income								
Less than €1500	289	59%	535	56%^ab^	311	57%	396	54%
€1500–€2999	281	59%	587	57%^a^	297	55%	318	50%
€3000 or more	221	53%	380	51%^b^	147	55%	124	44%
Prefer not to say/Don't know	108	58%	151	48% ^ab^	145	46%	81*	42%
Generation								
Generation Z (25 and under)	140	61%	199	71%^a^	125	46%^a^	122	58%
Millennials (26–41)	212	57%	413	53%^b^	221	52%^a^	241	48%
Younger Millennials (26–32)	93*	63%	179	63%^ab^	85*	42%^a^	99*	47%
Older Millennials (33–41)	119	51%	234	46%^b^	136	58%^ab^	142	49%
Generation X (42–57)	240	54%	457	49%^b^	290	53% ^a^	299	48%
Baby Boomers (58–76)	291	59%	543	54%^b^	248	62% ^b^	239	51%
Swing Generation/World War II (77+)	16*	—	41*	—	16*	—	18*	—

*Note:* Data percentages are not displayed for groups with fewer than 75 respondents, as the sample size is too low to provide meaningful insights and is represented as (−). Proportion Representation: Percentages reported in the tables are calculated within each socioeconomic group and represent the distribution of responses within these groups, rather than the overall percentage of the total population answering the question. For superscript: a, b: Different letters in the same column indicate statistically significant differences (*p* < 0.05); ab: Indicates no significant difference from both “a” and “b” values. Meanwhile, for superscript: x, y: Different letters in the same row indicate statistically significant differences (*p* < 0.05); xy: indicates no significant differences from both “x” and “y” values.

^1^ Only respondents who answered “Yes” to the previous question in Table 1 (Do you eat red meat (e.g., beef, pork, lamb, game) or poultry?) were included in the analysis for this question.

* Small sub‐sample: 75 to 100 is a low base size.

The analysis also revealed that females were significantly more likely than males to limit or reduce their meat intake over the previous 6 months (Table 2). However, no significant differences were observed among employment categories regarding the reduction of meat intake in any of the four countries.

When examining household income levels, consumers with higher incomes were generally less likely to limit or reduce their meat intake compared to lower‐income groups (Table 2). This trend was particularly significant in Germany, where income disparities appeared to influence meat reduction behaviors.

Generational patterns varied across countries. In Germany, Generation Z (25 years and younger) showed the highest tendency to reduce or limit meat consumption, with significant differences compared to older generations (Table 2). Conversely, in Italy, Baby Boomers reported higher percentages of meat reduction, differing significantly from younger generations. In France and Spain, no significant generational differences were observed regarding the limitation of meat intake.

### 3.3 Interest in Reducing Meat Consumption

Table 3 presents the findings on consumer interest in limiting or reducing meat intake in the future, focusing on those who had not previously restricted their consumption of red meat or poultry (as shown in Table 2). Overall, a small percentage of consumers in this group expressed interest (by answering “Yes”). Italy reported the highest interest (34%), followed by France (25%), Spain (20%), and Germany (18%) (Table 3).

**TABLE 3:** Interest in reducing meat consumption (asked of internet users aged 16+ who have not limited or reduced the amount of red meat/poultry they eat).

Would you be interested in limiting/reducing the amount of red meat/poultry you eat in the future?^1^	France (*n* = 382)		Germany (*n* = 752)		Italy (*n* = 410)		Spain (*n* = 457)	
All	Yes = 94 (25%)^xy^		Yes = 139 (18%)^y^		Yes = 139 (34%)^x^		Yes = 91 (20%)^y^	
	No = 288 (75%)		No = 613 (82%)		No = 271 (66%)		No = 366 (80%)	
Gender	*n*	%	*n*	%	*n*	%	*n*	%
Male	224	18%^a^	442	19%	227	37%	234	21%
Female	158	34%^b^	308	17%	183	30%	223	19%
Others	0	—	2	—	0	—	0	—
Employment								
Full‐time	171	27%	324	19%	174	31%	221	20%
Part‐time	43*	—	117	28%	75*	37%	55%	—
Not working	168	22%	311	15%	161	35%	181	20%
Net monthly household income								
Less than €1500	118	25%	235	17%	133	39%	183	25%^a^
€1500–€2999	116	21%	251	16%	133	35%	158	16%^b^
€3000 or more	103	28%	188	21%	66*	—	69*	—
Prefer not to say/Don't know	45*	—	78*	23%	78*	21%	47*	—
Generation								
Generation Z (25 and under)	54*	—	57*	—	67*	—	51*	—
Millennials (26–41)	92*	32%^a^	194	25%^a^	106	36%	125	20%
Younger Millennials (26–32)	34*	—	67*	—	49*	—	52*	—
Older Millennials (33–41)	58*	—	127	24% ^a^	57*	—	73*	—
Generation X (42–57)	110	22%^ab^	232	16% ^a^	136	26%	156	18%
Baby Boomers (58–76)	119	18%^b^	250	9%^b^	95*	31%	117	21%
Swing Generation/World War II (77+)	7*	—	19*	—	6*	—	8*	—

*Note:* Data percentages are not displayed for groups with fewer than 75 respondents, as the sample size is too low to provide meaningful insights and is represented as (−). Proportion Representation: Percentages reported in the tables are calculated within each socioeconomic group and represent the distribution of responses within these groups, rather than the overall percentage of the total population answering the question. For superscript: a, b: Different letters in the same column indicate statistically significant differences (*p* < 0.05); ab: Indicates no significant difference from both “a” and “b” values. Meanwhile, for superscript: x, y: Different letters in the same row indicate statistically significant differences (*p* < 0.05); xy: indicates no significant differences from both “x” and “y” values.

^1^ Only respondents who answered “No” to the previous question in Table 2 (Have you limited/reduced the amount of red meat/poultry you have eaten in the last 6 months?) were included in the analysis for this question.

* Small sub‐sample: 75 to 100 is a low base size.

A country‐wide comparison shows that Italy demonstrated the highest interest in reducing future meat consumption among consumers who had not limited their meat intake in the past 6 months. This interest was statistically significantly higher compared to all other countries except France (Table 3).

Gender differences were significant only in France, where females expressed greater interest in reducing meat intake than males (Table 3). In other countries, no significant differences were observed, with males generally showing a slightly higher percentage of interest in reducing meat consumption than females. Employment categories did not exhibit statistically significant differences in interest levels across any of the surveyed countries.

In terms of net monthly household income, Spanish consumers with low incomes showed significantly higher interest in reducing their meat intake compared to those in the moderate‐income group (Table 3). However, no significant income‐related differences were observed in the other countries.

In terms of net monthly household income, Spanish consumers with low incomes showed significantly higher interest in reducing their meat intake compared to those in the moderate‐income group (Table 3). However, no significant income‐related differences were observed in the other countries.

### 3.4 Consumers' Perceived Benefits of Reducing Meat Consumption

The analysis of consumers' perspectives on the perceived benefits of reducing meat consumption suggests that economic and sustainability motives are the most influential factors driving changes in food choices and consumption behaviors among European consumers. However, perceptions of these benefits vary across countries. For instance, economic motives were rated more highly in France and Spain, while sustainability motives were more prominent in Germany and Italy (Table 4).

**TABLE 4:** Consumers' perceived benefit of reducing meat consumption (asked of internet users aged 16+).

Eating less meat…? Please select all that apply	France (*n* = 1000)^x^	Germany (*n* = 2000)^x^	Italy (*n* = 1000)^y^	Spain (*n* = 1000)^y^
… is a good way to save money (economic motive)	42%	40%	34%	31%
… is better for the environment (sustainability motive)	34%	43%	40%	30%
… helps to reduce the risk of disease (health motive)	21%	22%	35%	30%
… is ethically correct (sustainability motive)	20%	24%	23%	21%
… makes you feel good (wellness motive)	18%	25%	28%	22%
… helps to manage weight (wellness motive)	17%	18%	21%	27%
… gives you more energy (health motive)	13%	11%	9%	10%

*Note:* x, y: Different letters in the same row indicate statistically significant differences (*p* < 0.05); xy: indicates no significant differences from both “x” and “y” values.

Among the countries surveyed, France and Germany demonstrated the highest recognition of the benefits associated with eating less meat, with 42% and 40% of respondents, respectively, identifying these advantages. The difference between these two countries is not statistically significant. In contrast, Italy and Spain showed significantly lower recognition compared to both France and Germany (Table 4).

Further insights into the effects of income and generational differences on consumers' perceived benefits of reducing meat consumption are detailed in Sections 1 and 2 of the Data S1. The data reveal a clear trend: lower‐income groups primarily view reducing meat consumption as a way to save money and improve energy levels. Conversely, as income levels increase, consumers are more likely to perceive meat reduction as beneficial for the environment, a means of reducing disease risk, ethically correct, personally rewarding (enhancing well‐being), and helpful for weight management.

Generational differences also play a significant role in shaping perceptions. Older generations, particularly Baby Boomers, tend to see reducing meat consumption as a practical strategy for saving money. Younger generations, including Millennials and Generation Z, are more inclined to view meat reduction as environmentally beneficial, ethically correct, and personally fulfilling, contributing to their well‐being and weight management.

### 3.5 Meat Substitute Purchasing Behaviors of Consumers

Consumers were asked whether they had purchased any meat substitutes in the previous 6 months, and the results are summarized in Table 5. Approximately half of the population reported purchasing some type of meat substitute during this period. Among the total sample, consumers in Italy reported the highest percentage of meat substitute purchases (41%), followed by Spain (36%), Germany (32%), and France (29%). No significant gender differences were observed in any of the countries (Table 5).

**TABLE 5:** Meat substitute purchasing behavior of consumers (asked of internet users aged 16+).

Have you bought any meat substitutes in the last 6 months?	France (*n* = 1000)		Germany (*n* = 2000)		Italy (*n* = 1000)		Spain (*n* = 1000)	
All	Yes = 290 (29%)^y^		Yes = 640 (32%)^y^		Yes = 408 (41%)^x^		Yes = 358 (36%)^y^	
	No = 685 (69%)		No = 1292 (65%)		No = 553 (55%)		No = 605 (61%)	
	Don't know = 25 (3%)		Don't know = 68 (3%)		Don't know = 39 (4%)		Don't know = 37 (4%)	
Gender								
Male	477	30%	978	31%	487	39%	489	37%
Female	521	28%	1012	33%	512	42%	511	35%
Others	2*	—	10*	—	1*	—	0	—
Employment								
Full‐time	447	36%^a^	834	40%^a^	425	44%^a^	474	39%^a^
Part‐time	110	35%^a^	328	41%^a^	169	50%^a^	126	40%^a^
Not working	443	21%^b^	838	21%^b^	406	34%^b^	400	30%^b^
Net monthly household income								
Less than €1500	334	31%	641	27%^a^	358	39%	428	36%
€1500–€2999	315	31%	703	33%^b^	320	45%	344	36%
€3000 or more	233	30%	456	41%^b^	156	47%	135	41%
Prefer not to say/Don't know	118	14%	200	24%^ab^	166	31%	93*	26%
Generation								
Generation Z (25 and under)	160	53%^a^	269	57%^a^	142	58%^a^	130	55%^a^
Millennials (26–41)	243	40%^a^	507	43%^a^	246	48%^a^	256	44%^a^
Younger Millennials (26–32)	109	46%^a^	217	52%^a^	95*	53%^a^	106	53%^a^
Older Millennials (33–41)	134	36%^a^	290	37%^a^	151	46%^a^	150	37%^a^
Generation X (42–57)	268	26%^a^	547	28%^a^	321	38%^ab^	327	36%^a^
Baby Boomers (58–76)	313	12%^b^	627	17%^b^	274	31%^b^	268	21%^b^
Swing Generation/World War II (77+)	16*	—	50*	—	17*	—	19*	—

*Note:* Data percentages are not displayed for groups with fewer than 75 respondents, as the sample size is too low to provide meaningful insights and is represented as (−). Proportion Representation: Percentages reported in the tables are calculated within each socioeconomic group and represent the distribution of responses within these groups, rather than the overall percentage of the total population answering the question. For superscript: a, b: Different letters in the same column indicate statistically significant differences (*p* < 0.05); ab: Indicates no significant difference from both “a” and “b” values. Meanwhile, for superscript: x, y: Different letters in the same row indicate statistically significant differences (*p* < 0.05); xy: indicates no significant differences from both “x” and “y” values.

* Small sub‐sample: 75 to 100 is a low base size.

A cross‐country comparison revealed that Italy had a significantly higher proportion of respondents purchasing meat substitutes compared to France, Germany, and Spain (Table 5). Additionally, significant differences were observed across employment categories in all countries. Consumers who were employed full‐time or part‐time reported purchasing meat substitutes more frequently than those who were unemployed.

Household income also played a role in meat substitute purchasing behavior. Consumers with higher incomes were more likely to purchase meat substitutes than those with lower incomes (Table 5). However, this difference was statistically significant only in Germany, where higher‐income consumers showed a marked difference from their lower‐income counterparts.

Generational analysis highlighted that Baby Boomers had the lowest percentage of meat substitute purchases compared to other generations, with significant differences observed between Baby Boomers and younger cohorts, such as Millennials and Generation Z (Table 5).

### 3.6 Trends and Types of Alternative Protein as Meat Substitutes Consumed

The consumption patterns of various alternative protein sources as meat substitutes were analyzed across four European countries. The results reveal notable country‐specific preferences. Fish substitutes (e.g., fish fingers, tuna) emerged as the most popular category in France (17%), Italy (28%), and Spain (22%). In contrast, breaded or battered meat/poultry substitutes were the preferred choice in Germany, with 18% of respondents selecting this category (Table 6).

**TABLE 6:** Trends in consumption of alternative protein sources as meat substitutes (asked of internet users aged 16+).

Types of meat substitutes eaten	France (*n* = 1000)	Germany (*n* = 2000)	Italy (*n* = 1000)	Spain (*n* = 1000)
Meat‐free sliced cold cuts (e.g., ham, salami)	14%	16%	14%	18%
Meat‐free burgers/meatballs	13%	15%	26%	20%
Breaded/battered meat/poultry substitutes	13%	18%	19%	15%
Un‐breaded/un‐battered alternatives to whole cuts of meat/poultry	13%	9%	13%	20%
Meat‐free sausages	9%	12%	9%	10%
Meat‐free mince/pieces	8%	14%	10%	11%
Unprocessed pieces of tempeh/tofu/seitan	7%	10%	9%	10%
Other meat substitute products	4%	5%	5%	5%
Fish substitutes (e.g., fish fingers, tuna)	17%	12%	28%	22%
I have not eaten meat substitutes in the last 6 months	58%	58%	42%	48%

### 3.7 Factors Affecting Meat Substitutes Purchase

Consumers were asked to select up to three factors influencing their preferences when purchasing meat substitutes, with the results summarized in Table 7. Across all four countries surveyed, three factors consistently emerged as key considerations: local sourcing and production, the same taste and texture as meat, and all‐natural ingredients. These findings indicate that consumers generally prioritize attributes related to local production, familiar sensory qualities, and natural composition when choosing meat substitutes.

**TABLE 7:** Factors affecting preference in purchasing meat substitutes (asked of internet users aged 16+ who have bought meat substitutes in the last 6 months).

Which of the following would most make you choose one meat substitute product over another? Please select up to 3^a^	France (*n* = 290)	Germany (*n* = 640)	Italy (*n* = 408)	Spain (*n* = 358)
Sourced and produced locally	27%	21%	35%	23%
2 Same taste/texture as the meat	26%	30%	25%	28%
3 All natural ingredients	26%	28%	36%	37%
4 Brand specializes in meat‐free products	21%	20%	21%	23%
5 Nutritionally rich	21%	23%	26%	34%
6 High vegetable/pulse content	21%	24%	30%	26%
7 Versatility	18%	25%	18%	22%
8 Brand that also produces meat products	16%	21%	13%	19%
9 Environmental impact	16%	18%	18%	13%
10 None of these	7%	6%	5%	2%

^a^ Only respondents who answered “Yes” to the previous question in Table 5 (Have you bought any meat substitutes in the last 6 months?) were included in this analysis.

A closer examination of country‐specific preferences revealed notable differences. In Italy and Spain, there is a particularly strong preference for meat substitutes containing all‐natural ingredients, with 36% of Italian and 37% of Spanish respondents identifying this as the most important factor. Additionally, Spanish consumers place considerable value on products with high nutritional content, with 34% citing this as a key driver of their purchasing decisions. This highlights the significance of health‐related attributes in shaping marketing trends, particularly in Spain.

In Germany, consumers show a stronger preference for versatility in meat substitutes, with 25% selecting this attribute as the most important. This preference suggests that German consumers value products that can be used in a variety of dishes and cooking styles, reflecting a focus on adaptability in culinary applications. Meanwhile, in France, preferences are more evenly distributed across several factors. Local sourcing and production (27%) and all‐natural ingredients (26%) are among the top considerations, but French consumers appear to prioritize a balanced combination of attributes, suggesting a wider range of criteria for selecting meat substitutes.

Further analysis of the effect of income on factors influencing consumer preferences is presented in Section 3 of the Data S1. The findings show a general trend: as income levels increase, there is greater demand for meat substitutes with natural ingredients and nutritional richness. This trend is particularly pronounced in France, Germany, and Italy. However, Spain deviates from this pattern, as preferences for natural ingredients and nutritional richness do not increase as significantly with income. These results suggest that while higher‐income consumers in most regions place greater emphasis on quality and health attributes, cultural or market‐specific factors may influence consumer preferences in Spain.

## 4 Discussion

The results of this study highlight several key points that require thorough discussion and further investigation. Firstly, meat consumption remains high across countries. However, Germany stands out with the highest proportion of individuals identifying as “vegan” or “vegetarian” and also exhibits lower meat consumption compared to other countries. This trend may be attributed to an increasing awareness in German consumers of the environmental and ethical implications associated with the mass production of meat and its consumption (Koch et al. 2019). These findings align with a report published by the United States Department of Agriculture's (USDA) Foreign Agricultural Service (2023), which noted that Germany has the highest rate of vegetarianism among European countries, with over 1.5 million vegetarians in 2022. According to the survey, the majority of Germans, specifically 55%, are now identified as flexitarians due to their reduced meat intake.

Besides, a positive trend was observed across European countries, where over half of red meat or poultry consumers reported reducing their intake in the past 6 months. In particular, consumers in France, Germany, and Italy are more inclined to reduce their red meat and poultry consumption, with over half of respondents in each country indicating such efforts. In contrast, Spain has a comparatively lower percentage of consumers engaged in reducing meat intake compared to others. This aligns with some previous studies suggesting that Spanish consumers tend to consume beef or pork more frequently than their European counterparts, with 11% of Spanish respondents reporting they eat these meats more than three times per week, exceeding the European average (Smart Protein 2023). Furthermore, these findings are consistent with a report published by Smart Protein (2023), which shows that European consumers are increasingly motivated to reduce their meat intake; 51% of meat consumers in Europe reported a reduction in their annual meat consumption, up from 46% in 2021. Given that Spanish consumers are less motivated to reduce meat consumption than those in the other three countries, it is essential to understand how their attitudes and awareness differ and to explore strategies that could increase their motivation to limit meat intake.

Further, our findings indicated that individuals who have not yet reduced their meat consumption show a low level of interest in doing so in the future. This result suggests that certain barriers may exist for these consumers that are making them less inclined to take a step forward in reducing meat intake. Possible explanations for this low interest could include cultural or personal preferences, a lack of perceived benefits in reducing meat consumption, or insufficient awareness of alternative dietary options, all of which might influence their interest or motivation in reducing meat intake (Sanchez‐Sabate, Badilla‐Briones, and Sabaté 2019). Moreover, Onwezen et al. (2019) reported that some consumers strategically ignore the sustainability and health concerns centering around the issue of industrial production and the mass consumption of meat, either because they do not care or because they choose to ignore these alarming issues.

Additionally, while there is a noticeable interest among consumers regarding meat substitutes and alternative proteins, relatively low purchase percentages indicate that these products are not yet mainstream in consumer diets across various countries. A possible explanation for this might be the higher price and limited availability of meat alternative options, often confined to high‐end stores and specialty markets, as reported by Nezlek and Forestell (2022). These factors discourage consumers from purchasing meat alternatives due to accessibility constraints. Besides, taste, price, and convenience concerns have also been cited as barriers to adopting alternative protein sources or a vegetarian or vegan diet (Bryant 2019). Moreover, both non‐users and users of meat alternatives agree that, compared to meat, an ideal meat substitute should be more affordable, higher in protein and vitamins, and lower in calories (Hoek et al. 2011).

Besides, our study highlighted the significance of economic and sustainability considerations that play a significant role in nudging consumers and motivating them to reduce their meat purchases and consumption. This suggests that consumers' food choices and dietary habits are motivated not only by personal health concerns but also by broader considerations, such as environmental sustainability and economic savings when choosing the reasons for eating less meat. Apostolidis and McLeay (2016) also reported a similar finding and emphasized that price is one of the crucial factors in shaping consumer preferences toward reducing meat consumption and opting for meat substitutes and alternative protein sources. However, our findings differ from those of the recent study by Downs et al. (2024), which identified health and price as key factors influencing red meat reduction, while environmental sustainability and animal welfare were found to be less important considerations among US respondents.

One of the most fascinating findings of our study is the prevalence of fish and fish substitutes as popular and viable alternatives among consumers to supplement protein demand, rather than traditional meat. This trend potentially indicates a gradual shift toward more sustainable and health‐conscious dietary patterns across Europe. Consistent with prior studies (Culliford and Bradbury 2020; de Boer, Schösler, and Aiking 2020; Torrissen and Onozaka 2017), there is a consistent consumer preference for fish substitutes as alternative protein sources. Notably, the acceptance of fish as an alternative protein varies across countries, influenced by culinary habits, cultural preferences, and traditions. For instance, Italian consumers show a strong cultural preference for seafood and fish‐based alternatives (Gasco et al. 2020). In Spain, while fish consumption is widespread, its relatively high cost may limit its mass adoption as a meat substitute (Carlucci et al. 2015). Conversely, French consumers demonstrate lower acceptance of fish as a meat alternative (Melendrez‐Ruiz et al. 2019). Interestingly, German consumers diverge from this pattern. Though they show a decreasing trend in meat consumption, instead of opting for fish‐based alternative proteins, they prefer breaded or battered meat and poultry substitutes. These variations underscore the diversity of culinary preferences and dietary habits across European countries, highlighting how cultural and economic factors shape the acceptance of different alternative proteins like fish as a sustainable protein source (Cunha et al. 2018). However, there is a need to promote more responsible consumption. As the demand for fish products grows, concerns about sustainable fisheries are not as keenly perceived in Italy (Bonanomi et al. 2017).

Interestingly, we found three factors consistently chosen as the most contributing attributes influencing consumers' decisions to purchase meat substitutes. Specifically, local production, achieving the same taste and texture as meat, and all‐natural ingredients are considered crucial when selecting meat substitute options. These findings are supported by Román, Sánchez‐Siles, and Siegrist (2017) in their systematic review of 72 studies, which indicates that consumers show a stronger preference for natural items in foods compared to medicines, and that perceived naturalness is a crucial factor for the acceptance of foods and food technologies. Furthermore, a study conducted by Paul Fesenfeld et al. (2024) suggests that texture has a slightly more positive influence than taste, particularly in the context of desired canteen offerings.

Regarding gender‐based differences, it was found that females have comparatively less consumption of red meat and demonstrate greater interest in limiting their meat intake compared to the male consumer base. This is not surprising, as previous studies have reported that gender influences meat consumption habits, and that women consume less meat than men (Downs et al. 2024; Hayley, Zinkiewicz, and Hardiman 2015; Prättälä et al. 2006; Rosenfeld and Tomiyama 2021). In addition, girls and women seem to choose a semi‐vegetarian diet making them flexitarians, whereas boys and men tend to follow omnivorous diets (Smart Protein 2023). Veganism also seems to be skewed by gender, with it being more common among women and girls (Smart Protein 2023).

Our analysis also uncovered a notable correlation between income levels and meat consumption patterns among consumers. Specifically, individuals with higher incomes tend to consume more meat than those with lower incomes. This trend may be attributed to the increased purchasing power associated with higher incomes, which enables these individuals to afford meat‐rich diets more frequently. This aligns with previous studies that reported similar patterns, indicating that higher‐income individuals tend to consume more meat, while those with lower incomes exhibit a stronger interest in reducing their meat intake (Klink et al. 2022; Liu et al. 2023; Macdiarmid, Douglas, and Campbell 2016). However, contradictory behavior was also observed where consumers with a comparatively higher income purchased more meat alternatives in recent months than individuals with less income. This phenomenon is also associated with the assumption that rich people have higher purchasing power and the ability to afford pricier or more costly meat alternatives (Gómez‐Luciano et al. 2019; Nezlek and Forestell 2022). This suggests that socioeconomic factors play a significant role in shaping dietary behaviors, with income disparities influencing access to and preferences for various food options (Lee et al. 2021; Li et al. 2023).

On the other hand, in terms of generation‐based differences, it is interesting to see that the Baby Boomer generation tends to consume more meat and shows less interest in limiting their intake, while the younger generation exhibits contrasting behavior, with a tendency toward limited meat consumption and a greater interest in reducing it. Research on generational attitudes toward meat consumption and meat alternatives has uncovered a complex landscape. For instance, Jürkenbeck, Spiller, and Schulze (2021) found that about half of younger individuals are highly aware of climate change, and those with heightened climate awareness are more likely to make climate‐friendly dietary choices. Moreover, one possible explanation for this generational divide could be the differing levels of awareness of the environmental impact of meat consumption which was also reflected in some other studies (Di Novi and Marenzi 2022; Diprose et al. 2019). It is conceivable that younger generations, who have grown up in a time of increased environmental consciousness and sustainability concerns, are more aware of the ecological and health effects of meat production and consumption (Jürkenbeck, Spiller, and Schulze 2021). This awareness may motivate them to adopt more plant‐based or vegan or flexitarian diets to reduce their environmental footprint (Venter de Villiers, Cheng, and Truter 2024). Conversely, older generations, such as Baby Boomers, may have been less exposed to, or less concerned about, environmental and health issues related to industrial meat production and meat consumption during their formative years (Diprose et al. 2019). Consequently, they may maintain traditional dietary habits characterized by higher meat consumption without the same level of consideration for healthier dietary habits and environmental sustainability.

### 4.1 Implications

This study presents several key implications for consumers, policymakers, producers and businesses regarding current patterns in the acceptance and consumption of meat substitutes. Firstly, there is a growing trend among Western European consumers to reduce meat consumption and transition to more sustainable and healthier alternative proteins. This shift is expected to have substantial positive effects on environmental sustainability and public health, changing dietary habits and impacting food producers and guiding policy decisions. Secondly, our findings emphasize the importance of educating consumers about the economic and environmental benefits of reducing meat intake. To encourage this shift, public awareness campaigns emphasizing the health and environmental benefits of incorporating alternative proteins into diets could be implemented across Europe. These initiatives would help consumers understand the impact of dietary habits on personal health and the environment, empowering them to make informed choices and potentially reducing overall meat consumption. To further promote meat alternatives, well‐planned marketing strategies should focus on communicating their nutritional, environmental, and health benefits on a wider scale, as suggested by Thavamani, Sferra, and Sankararaman (2020). For businesses, adapting to these changing preferences by expanding their offerings of affordable meat alternative options could meet the rising demand. Besides, our results indicated that fish is a particularly popular meat substitute, suggesting an opportunity to broaden the meat alternative product lines with more fish‐based products. Further, marketing strategies should emphasize attributes such as natural ingredients, taste and texture similarity to meat, and local production when promoting products, as these factors are likely to influence consumer preferences to purchase meat substitutes. Overall, this study found that consumers still exhibit low purchasing behavior toward meat substitute products, underscoring the need to increase the availability and affordability of alternative proteins in the market. On the other hand, promotional and marketing strategies should be tailored to address current trends and consumer preferences to maximize market penetration and consumer acceptance of meat alternative products.

### 4.2 Limitations and Future Research

This study has several limitations. First, the outcomes are hypothetical and subject to hypothetical bias. Respondents may not have an accurate understanding of their meat consumption levels, and social desirability bias may influence self‐reporting—leading individuals to provide answers they believe are socially acceptable rather than reflecting their actual behavior. Second, although the sample size was relatively large, it may not fully represent the diversity of dietary habits and attitudes across all European populations due to varied cultural backgrounds and culinary practices. The results may not be generalizable to the entire European population, and variations in attitudes and behaviors within each country may not have been fully captured. Consequently, future research should examine dietary habits and attitudes in Eastern European regions to generate a more inclusive scenario of European consumers' acceptance and purchase behavior regarding meat alternative products. This approach will provide a more comprehensive understanding of the cultural and regional differences in dietary practices within European countries. In addition, given the exponential increase in meat consumption observed in Asia over the past five decades, as highlighted by Bonnet et al. (2020), it is imperative to expand the scope of this research to include Asian regions. This would provide a wide‐ranging and comprehensive overview of global dietary trends and allow for comparisons between European and Asian populations, which could also be replicated in other continents. Finally, research analyzing the impact of reducing meat consumption on markets and farming activities is needed to better understand the implications of this shift in consumer preferences.

## 5 Conclusion

The study highlights the ongoing transformation of European consumers' dietary habits, with an increasing interest in reducing their meat consumption and embracing alternative protein sources. Overall, more than 50% of Western European consumers reported consuming limited amounts of red meat. The study also found that less than a third of the respondents who have not reduced their meat intake are interested in doing so. Interestingly, the findings suggest the importance of economic and sustainability motives associated with eating less meat, which influence consumers' perceptions and purchase decisions. Moreover, local sourcing/production, natural ingredients and the same taste/texture as meat were rated as the main reasons for purchasing meat substitutes. Therefore, food industries and businesses should invest more in developing products that will meet consumers' needs for sustainable and healthier diets in alignment with their changing preferences.

## Author Contributions

**Muhammad Adzran Che Mustapa:** conceptualization (lead), data curation (lead), formal analysis (lead), methodology (lead), visualization (lead), writing – original draft (lead). **Yasmina Baba:** validation (supporting), visualization (supporting), writing – review and editing (equal). **Smita Dash Baishakhy:** methodology (supporting), validation (supporting), writing – review and editing (equal). **Zein Kallas:** conceptualization (lead), funding acquisition (lead), supervision (lead), validation (equal), writing – review and editing (equal).

## Conflicts of Interest

The authors declare no conflicts of interest.

## Supporting information


Data S1.

## Data Availability

The data that support the findings of this study are available on request from the corresponding author. The data are not publicly available due to privacy or ethical restrictions.

## References

[fsn34753-bib-0001] Andersson, J. , and K. Hannah . 2023. “To What Extent EU Regulations and Consumer Behavior Have Affected the Expansion of Alternative Proteins: A Comparison of the Plant‐Based and Cell‐Based Meat Markets.” http://uu.diva‐portal.org/smash/record.jsf?pid=diva2%3A1789445&dswid=3146.

[fsn34753-bib-0002] Apostolidis, C. , and F. McLeay . 2016. “Should We Stop Meating Like This? Reducing Meat Consumption Through Substitution.” Food Policy 65: 74–89. 10.1016/j.foodpol.2016.11.002.

[fsn34753-bib-0003] BCG . 2021. “Alternative‐Protein Market to Reach At Least $290 Billion by 2035.” https://www.bcg.com/press/23march2021‐alternative‐protein‐market‐reach‐290‐billion‐by‐2035.

[fsn34753-bib-0004] Bellavia, A. , F. Stilling , and A. Wolk . 2016. “High Red Meat Intake and All‐Cause Cardiovascular and Cancer Mortality: Is the Risk Modified by Fruit and Vegetable Intake?” American Journal of Clinical Nutrition 104, no. 4: 1137–1143. 10.3945/ajcn.116.135335. 27557655

[fsn34753-bib-0005] Binnie, M. A. , K. Barlow , V. Johnson , and C. Harrison . 2014. “Red Meats: Time for a Paradigm Shift in Dietary Advice.” Meat Science 98, no. 3: 445–451. 10.1016/j.meatsci.2014.06.024. 25041653

[fsn34753-bib-0006] Bonanomi, S. , A. Colombelli , L. Malvarosa , M. Cozzolino , and A. Sala . 2017. “Towards the Introduction of Sustainable Fishery Products: The Bid of a Major Italian Retailer.” Sustainability 9, no. 3: 438. 10.3390/su9030438.

[fsn34753-bib-0007] Bonnet, C. , Z. Bouamra‐Mechemache , V. Réquillart , and N. Treich . 2020. “Viewpoint: Regulating Meat Consumption to Improve Health, the Environment and Animal Welfare.” Food Policy 97: 101847. 10.1016/j.foodpol.2020.101847.

[fsn34753-bib-0008] Bryant, C. J. 2019. “We Can't Keep Meating Like This: Attitudes Towards Vegetarian and Vegan Diets in the United Kingdom.” Sustainability 11, no. 23: 6844. 10.3390/su11236844.

[fsn34753-bib-0009] Bryant, C. J. 2022. “Plant‐Based Animal Product Alternatives Are Healthier and More Environmentally Sustainable Than Animal Products.” Future Foods 6: 100174. 10.1016/j.fufo.2022.100174.

[fsn34753-bib-0010] Cai, J. , S. Wang , Y. Li , et al. 2024. “Industrialization Progress and Challenges of Cultivated Meat.” Journal of Future Foods 4, no. 2: 119–127. 10.1016/j.jfutfo.2023.06.002.

[fsn34753-bib-0011] Carlucci, D. , G. Nocella , B. De Devitiis , R. Viscecchia , F. Bimbo , and G. Nardone . 2015. “Consumer Purchasing Behaviour Towards Fish and Seafood Products. Patterns and Insights From a Sample of International Studies.” Appetite 84: 212–227. 25453592 10.1016/j.appet.2014.10.008

[fsn34753-bib-0012] Culliford, A. , and J. Bradbury . 2020. “A Cross‐Sectional Survey of the Readiness of Consumers to Adopt an Environmentally Sustainable Diet.” Nutrition Journal 19, no. 1: 138. 10.1186/s12937-020-00644-7. 33298065 PMC7727219

[fsn34753-bib-0013] Cunha, L. M. , D. Cabral , A. P. Moura , and M. D. V. de Almeida . 2018. “Application of the Food Choice Questionnaire Across Cultures: Systematic Review of Cross‐Cultural and Single Country Studies.” Food Quality and Preference 64: 21–36. 10.1016/j.foodqual.2017.10.007.

[fsn34753-bib-0014] de Bakker, E. , and H. Dagevos . 2012. “Reducing Meat Consumption in Today's Consumer Society: Questioning the Citizen‐Consumer Gap.” Journal of Agricultural and Environmental Ethics 25, no. 6: 877–894. 10.1007/s10806-011-9345-z.

[fsn34753-bib-0015] de Boer, J. , H. Schösler , and H. Aiking . 2020. “Fish as an Alternative Protein–A Consumer‐Oriented Perspective on Its Role in a Transition Towards More Healthy and Sustainable Diets.” Appetite 152: 104721. 10.1016/j.appet.2020.104721. 32343989

[fsn34753-bib-0016] Demeyer, D. , B. Mertens , S. De Smet , and M. Ulens . 2016. “Mechanisms Linking Colorectal Cancer to the Consumption of (Processed) Red Meat: A Review.” Critical Reviews in Food Science and Nutrition 56, no. 16: 2747–2766. 10.1080/10408398.2013.873886. 25975275

[fsn34753-bib-0017] Di Novi, C. , and A. Marenzi . 2022. “Improving Health and Sustainability: Patterns of Red and Processed Meat Consumption Across Generations.” Health Policy 126, no. 12: 1324–1330. 10.1016/j.healthpol.2022.10.006. 36266131 PMC9709574

[fsn34753-bib-0018] Diprose, K. , G. Valentine , R. M. Vanderbeck , C. Liu , and K. McQuaid . 2019. “Building Common Cause Towards Sustainable Consumption: A Cross‐Generational Perspective.” Environment and Planning E: Nature and Space 2, no. 2: 203–228. 10.1177/2514848619834845.

[fsn34753-bib-0019] Downs, S. M. , E. V. Merchant , J. Sackey , E. L. Fox , C. Davis , and J. Fanzo . 2024. “Sustainability Considerations Are Not Influencing Meat Consumption in the US.” Appetite 203: 107667. 10.1016/j.appet.2024.107667. 39243869

[fsn34753-bib-0020] Fonseca, R. P. , and R. Sanchez‐Sabate . 2022. “Consumers' Attitudes Towards Animal Suffering: A Systematic Review on Awareness, Willingness and Dietary Change.” International Journal of Environmental Research and Public Health 19, no. 23: 16372. 10.3390/ijerph192316372. 36498444 PMC9741386

[fsn34753-bib-0021] Gasco, L. , G. Acuti , P. Bani , et al. 2020. “Insect and Fish By‐Products as Sustainable Alternatives to Conventional Animal Proteins in Animal Nutrition.” Italian Journal of Animal Science 19, no. 1: 360–372. 10.1080/1828051X.2020.1743209.

[fsn34753-bib-0022] GFI . 2022. “Market Insights on European Plant‐Based Sales 2020–2022.” https://gfieurope.org/market‐insights‐on‐european‐plant‐based‐sales‐2020‐2022/.

[fsn34753-bib-0023] Gómez‐Luciano, C. A. , L. K. de Aguiar , F. Vriesekoop , and B. Urbano . 2019. “Consumers' Willingness to Purchase Three Alternatives to Meat Proteins in the United Kingdom, Spain, Brazil and The Dominican Republic.” Food Quality and Preference 78: 103732. 10.1016/j.foodqual.2019.103732.

[fsn34753-bib-0024] Graça, J. , M. M. Calheiros , and A. Oliveira . 2014. “Moral Disengagement in Harmful but Cherished Food Practices? An Exploration Into the Case of Meat.” Journal of Agricultural and Environmental Ethics 27, no. 5: 749–765. 10.1007/s10806-014-9488-9.

[fsn34753-bib-0025] Graça, J. , C. A. Godinho , and M. Truninger . 2019. “Reducing Meat Consumption and Following Plant‐Based Diets: Current Evidence and Future Directions to Inform Integrated Transitions.” Trends in Food Science & Technology 91: 380–390. 10.1016/j.tifs.2019.07.046.

[fsn34753-bib-0026] Guenther, P. M. , H. H. Jensen , S. P. Batres‐Marquez , and C.‐F. Chen . 2005. “Sociodemographic, Knowledge, and Attitudinal Factors Related to Meat Consumption in the United States.” Journal of the American Dietetic Association 105, no. 8: 1266–1274. 10.1016/j.jada.2005.05.014. 16182644

[fsn34753-bib-0027] Hartmann, C. , P. Furtwaengler , and M. Siegrist . 2022. “Consumers' Evaluation of the Environmental Friendliness, Healthiness and Naturalness of Meat, Meat Substitutes, and Other Protein‐Rich Foods.” Food Quality and Preference 97: 104486. 10.1016/j.foodqual.2021.104486.

[fsn34753-bib-0028] Hartmann, C. , and M. Siegrist . 2017. “Consumer Perception and Behaviour Regarding Sustainable Protein Consumption: A Systematic Review.” Trends in Food Science & Technology 61: 11–25. 10.1016/j.tifs.2016.12.006.

[fsn34753-bib-0029] Hartmann, C. , and M. Siegrist . 2020. “Our Daily Meat: Justification, Moral Evaluation and Willingness to Substitute.” Food Quality and Preference 80: 103799. 10.1016/j.foodqual.2019.103799.

[fsn34753-bib-0030] Hayley, A. , L. Zinkiewicz , and K. Hardiman . 2015. “Values, Attitudes, and Frequency of Meat Consumption. Predicting Meat‐Reduced Diet in Australians.” Appetite 84: 98–106. 10.1016/j.appet.2014.10.002. 25312749

[fsn34753-bib-0031] Henn, K. , S. Bøye Olsen , H. Goddyn , and W. L. P. Bredie . 2022. “Willingness to Replace Animal‐Based Products With Pulses Among Consumers in Different European Countries.” Food Research International 157: 111403. 10.1016/j.foodres.2022.111403. 35761657

[fsn34753-bib-0032] Hoek, A. C. , P. A. Luning , P. Weijzen , W. Engels , F. J. Kok , and C. Graaf . 2011. “Replacement of Meat by Meat Substitutes. A Survey on Person‐ and Product‐Related Factors in Consumer Acceptance.” Appetite 56: 662–673. 10.1016/j.appet.2011.02.001. 21315123

[fsn34753-bib-0033] Jürkenbeck, K. , A. Spiller , and M. Schulze . 2021. “Climate Change Awareness of the Young Generation and Its Impact on Their Diet.” Cleaner and Responsible Consumption 3: 100041. 10.1016/j.clrc.2021.100041.

[fsn34753-bib-0034] Kemper, J. A. 2020. “Motivations, Barriers, and Strategies for Meat Reduction at Different Family Lifecycle Stages.” Appetite 150: 104644. 10.1016/j.appet.2020.104644. 32109523

[fsn34753-bib-0035] Klink, U. , J. Mata , R. Frank , and B. Schüz . 2022. “Socioeconomic Differences in Animal Food Consumption: Education Rather Than Income Makes a Difference.” Frontiers in Nutrition 9: 993379. 10.3389/fnut.2022.993379. 36407520 PMC9668869

[fsn34753-bib-0036] Koch, F. , T. Heuer , C. Krems , and E. Claupein . 2019. “Meat Consumers and Non‐Meat Consumers in Germany: A Characterisation Based on Results of the German National Nutrition Survey II.” Journal of Nutritional Science 8: e21. 10.1017/jns.2019.17. 31217969 PMC6558667

[fsn34753-bib-0037] Kopplin, C. S. , and T. M. Rausch . 2022. “Above and Beyond Meat: The Role of Consumers' Dietary Behavior for the Purchase of Plant‐Based Food Substitutes.” Review of Managerial Science 16, no. 5: 1335–1364. 10.1007/s11846-021-00480-x.

[fsn34753-bib-0038] Larsson, S. C. , and N. Orsini . 2013. “Red Meat and Processed Meat Consumption and All‐Cause Mortality: A Meta‐Analysis.” American Journal of Epidemiology 179, no. 3: 282–289. 10.1093/aje/kwt261. 24148709

[fsn34753-bib-0039] Lee, A. , D. Patay , L.‐M. Herron , E. Parnell Harrison , and M. Lewis . 2021. “Affordability of Current, and Healthy, More Equitable, Sustainable Diets by Area of Socioeconomic Disadvantage and Remoteness in Queensland: Insights Into Food Choice.” International Journal for Equity in Health 20, no. 1: 153. 10.1186/s12939-021-01481-8. 34193163 PMC8243618

[fsn34753-bib-0040] Lemken, D. , A. Spiller , and B. Schulze‐Ehlers . 2019. “More Room for Legume—Consumer Acceptance of Meat Substitution With Classic, Processed and Meat‐Resembling Legume Products.” Appetite 143: 104412. 10.1016/j.appet.2019.104412. 31445994

[fsn34753-bib-0041] Lentz, G. , S. Connelly , M. Mirosa , and T. Jowett . 2018. “Gauging Attitudes and Behaviours: Meat Consumption and Potential Reduction.” Appetite 127: 230–241. 10.1016/j.appet.2018.04.015. 29751024

[fsn34753-bib-0042] Lescinsky, H. , A. Afshin , C. Ashbaugh , et al. 2022. “Health Effects Associated With Consumption of Unprocessed Red Meat: A Burden of Proof Study.” Nature Medicine 28, no. 10: 2075–2082. 10.1038/s41591-022-01968-z. PMC9556326 36216940

[fsn34753-bib-0043] Li, C. , T. R. P. Bishop , F. Imamura , et al. 2024. “Meat Consumption and Incident Type 2 Diabetes: An Individual‐Participant Federated Meta‐Analysis of 1.97 Million Adults With 100 000 Incident Cases From 31 Cohorts in 20 Countries.” Lancet Diabetes & Endocrinology 12, no. 9: 619–630. 10.1016/S2213-8587(24)00179-7. 39174161

[fsn34753-bib-0044] Li, J. , C. Silver , M. I. Gómez , M. Milstein , and G. Sogari . 2023. “Factors Influencing Consumer Purchase Intent for Meat and Meat Substitutes.” Future Foods 7: 100236. 10.1016/j.fufo.2023.100236.

[fsn34753-bib-0045] Liu, J. , S. Chriki , M. Kombolo , et al. 2023. “Consumer Perception of the Challenges Facing Livestock Production and Meat Consumption.” Meat Science 200: 109144. 10.1016/j.meatsci.2023.109144. 36863253

[fsn34753-bib-0046] Macdiarmid, J. I. , F. Douglas , and J. Campbell . 2016. “Eating Like There's no Tomorrow: Public Awareness of the Environmental Impact of Food and Reluctance to Eat Less Meat as Part of a Sustainable Diet.” Appetite 96: 487–493. 10.1016/j.appet.2015.10.011. 26476397

[fsn34753-bib-0047] Matos, S. , E. Viardot , B. K. Sovacool , F. W. Geels , and Y. Xiong . 2022. “Innovation and Climate Change: A Review and Introduction to the Special Issue.” Technovation 117: 102612. 10.1016/j.technovation.2022.102612.

[fsn34753-bib-0048] McAfee, A. J. , E. M. McSorley , G. J. Cuskelly , et al. 2010. “Red Meat Consumption: An Overview of the Risks and Benefits.” Meat Science 84, no. 1: 1–13. 10.1016/j.meatsci.2009.08.029. 20374748

[fsn34753-bib-0049] Melendrez‐Ruiz, J. , S. Chambaron , Q. Buatois , S. Monnery‐Patris , and G. Arvisenet . 2019. “A Central Place for Meat, but What About Pulses? Studying French Consumers' Representations of Main Dish Structure, Using an Indirect Approach.” Food Research International 123: 790–800. 10.1016/j.foodres.2019.06.004. 31285029

[fsn34753-bib-0050] Michel, F. , C. Hartmann , and M. Siegrist . 2021. “Consumers' Associations, Perceptions and Acceptance of Meat and Plant‐Based Meat Alternatives.” Food Quality and Preference 87: 104063. 10.1016/j.foodqual.2020.104063.

[fsn34753-bib-0051] Murray, C. J. L. , A. Y. Aravkin , and P. Zheng . 2020. “Global Burden of 87 Risk Factors in 204 Countries and Territories, 1990‐2019: A Systematic Analysis for the Global Burden of Disease Study 2019.” Lancet 396, no. 10258: 1223–1249. 10.1016/S0140-6736(20)30752-2. 33069327 PMC7566194

[fsn34753-bib-0052] Nezlek, J. B. , and C. A. Forestell . 2022. “Meat Substitutes: Current Status, Potential Benefits, and Remaining Challenges.” Current Opinion in Food Science 47: 100890. 10.1016/j.cofs.2022.100890.

[fsn34753-bib-0053] Onwezen, M. C. , J. van den Puttelaar , M. C. D. Verain , and T. Veldkamp . 2019. “Consumer Acceptance of Insects as Food and Feed: The Relevance of Affective Factors.” Food Quality and Preference 77: 51–63. 10.1016/j.foodqual.2019.04.011.

[fsn34753-bib-0054] Paul Fesenfeld, L. , N. Zeiske , M. Maier , M. Rachelle Gallmann , E. Van der Werff , and L. Steg . 2024. “Tasting and Labeling Meat Substitute Products Can Affect Consumers' Product Evaluations and Preferences.” Food Quality and Preference 118: 105184. 10.1016/j.foodqual.2024.105184.

[fsn34753-bib-0055] Petersen, T. , M. Hartmann , and S. Hirsch . 2021. “Which Meat (Substitute) to Buy? Is Front of Package Information Reliable to Identify the Healthier and More Natural Choice?” Food Quality and Preference 94: 104298. 10.1016/j.foodqual.2021.104298.

[fsn34753-bib-0056] Petersen, T. , and S. Hirsch . 2023. “Comparing Meat and Meat Alternatives: An Analysis of Nutrient Quality in Five European Countries.” Public Health Nutrition 26, no. 12: 3349–3358. 10.1017/S1368980023001945. 37800339 PMC10755401

[fsn34753-bib-0057] Prättälä, R. , L. Paalanen , D. Grinberga , V. Helasoja , A. Kasmel , and J. Petkeviciene . 2006. “Gender Differences in the Consumption of Meat, Fruit and Vegetables Are Similar in Finland and the Baltic Countries.” European Journal of Public Health 17, no. 5: 520–525. 10.1093/eurpub/ckl265. 17194710

[fsn34753-bib-0058] Ramos‐Souza, C. , D. H. Bandoni , A. P. A. Bragotto , and V. V. De Rosso . 2023. “Risk Assessment of Azo Dyes as Food Additives: Revision and Discussion of Data Gaps Toward Their Improvement.” Comprehensive Reviews in Food Science and Food Safety 22, no. 1: 380–407. 10.1111/1541-4337.13072. 36374221

[fsn34753-bib-0059] Report, M. A. 2024. “Meat Substitutes: Market Size & Trends.” https://www.grandviewresearch.com/industry‐analysis/meat‐substitutes‐market.

[fsn34753-bib-0060] Román, S. , L. M. Sánchez‐Siles , and M. Siegrist . 2017. “The Importance of Food Naturalness for Consumers: Results of a Systematic Review.” Trends in Food Science & Technology 67: 44–57. 10.1016/j.tifs.2017.06.010.

[fsn34753-bib-0061] Rosenfeld, D. L. , and A. J. Tomiyama . 2021. “Gender Differences in Meat Consumption and Openness to Vegetarianism.” Appetite 166: 105475. 10.1016/j.appet.2021.105475. 34166748

[fsn34753-bib-0062] Sanchez‐Sabate, R. , Y. Badilla‐Briones , and J. Sabaté . 2019. “Understanding Attitudes Towards Reducing Meat Consumption for Environmental Reasons. A Qualitative Synthesis Review.” Sustainability 11, no. 22: 6295.

[fsn34753-bib-0063] Service, U.F.A . 2023. “Plant‐Based Food Goes Mainstream in Germany (GM2023‐0002).” https://apps.fas.usda.gov/newgainapi/api/Report/DownloadReportByFileName?fileName=Plant‐Based%20Food%20Goes%20Mainstream%20in%20Germany_Berlin_Germany_GM2023‐0002.pdf.

[fsn34753-bib-0064] Siegrist, M. , F. Michel , and C. Hartmann . 2024. “The Shift From Meat to Plant‐Based Proteins: Consumers and Public Policy.” Current Opinion in Food Science 58: 101182. 10.1016/j.cofs.2024.101182.

[fsn34753-bib-0065] Smart Protein . 2023. “Evolving Appetites: An In‐Depth Look at European Attitudes Towards Plant‐Based Eating.” https://smartproteinproject.eu/european‐attitudes‐towards‐plant‐based‐eating/.

[fsn34753-bib-0066] Springmann, M. , K. Wiebe , D. Mason‐D'Croz , T. B. Sulser , M. Rayner , and P. Scarborough . 2018. “Health and Nutritional Aspects of Sustainable Diet Strategies and Their Association With Environmental Impacts: A Global Modelling Analysis With Country‐Level Detail.” Lancet Planetary Health 2, no. 10: e451–e461. 10.1016/S2542-5196(18)30206-7. 30318102 PMC6182055

[fsn34753-bib-0067] Steinfeld, H. 2006. “Livestock's Long Shadow: Environmental Issues and Options.” Food and Agriculture Organization of the United Nations 24: 1–390. https://www.fao.org/publications/card/en/c/9655af93‐7f88‐58fc‐84e8‐d70a9a4d8bec/.

[fsn34753-bib-0068] Tarrega, A. , A. Rizo , A. Murciano , L. Laguna , and S. Fiszman . 2020. “Are Mixed Meat and Vegetable Protein Products Good Alternatives for Reducing Meat Consumption? A Case Study With Burgers.” Current Research in Food Science 3: 30–40. 10.1016/j.crfs.2020.02.003. 32914118 PMC7473368

[fsn34753-bib-0069] Thavamani, A. , T. J. Sferra , and S. Sankararaman . 2020. “Meet the Meat Alternatives: The Value of Alternative Protein Sources.” Current Nutrition Reports 9, no. 4: 346–355. 10.1007/s13668-020-00341-1. 33151486

[fsn34753-bib-0070] Tonheim, L. E. , E. Austad , L. E. Torheim , and S. Henjum . 2022. “Plant‐Based Meat and Dairy Substitutes on the Norwegian Market: Comparing Macronutrient Content in Substitutes With Equivalent Meat and Dairy Products.” Journal of Nutritional Science 11: e9. 10.1017/jns.2022.6. 35291275 PMC8889083

[fsn34753-bib-0071] Torrissen, J. K. , and Y. Onozaka . 2017. “Comparing Fish to Meat: Perceived Qualities by Food Lifestyle Segments.” Aquaculture Economics & Management 21, no. 1: 44–70. 10.1080/13657305.2017.1265022.

[fsn34753-bib-0072] Tosun, P. , M. Yanar , S. Sezgin , and N. Uray . 2021. “Meat Substitutes in Sustainability Context: A Content Analysis of Consumer Attitudes.” Journal of International Food & Agribusiness Marketing 33, no. 5: 541–563. 10.1080/08974438.2020.1840475.

[fsn34753-bib-0073] Tyndall, S. M. , G. R. Maloney , M. B. Cole , N. G. Hazell , and M. A. Augustin . 2024. “Critical Food and Nutrition Science Challenges for Plant‐Based Meat Alternative Products.” Critical Reviews in Food Science and Nutrition 64, no. 3: 638–653. 10.1080/10408398.2022.2107994. 35972071

[fsn34753-bib-0074] Venter de Villiers, M. , J. Cheng , and L. Truter . 2024. “The Shift Towards Plant‐Based Lifestyles: Factors Driving Young Consumers' Decisions to Choose Plant‐Based Food Products.” Sustainability 16, no. 20: 9022. 10.3390/su162090229022.

[fsn34753-bib-0075] Weis, T. 2013. “The Meat of the Global Food Crisis.” Journal of Peasant Studies 40, no. 1: 65–85. 10.1080/03066150.2012.752357.

[fsn34753-bib-0076] Zur, I. , and C. Klöckner . 2014. “Individual Motivations for Limiting Meat Consumption.” British Food Journal 116, no. 4: 629–642. 10.1108/BFJ-08-2012-0193.

