# Supplementary material for: Evolving Appetites: Current Evidence and Future Perspectives in Terms of Meat Substitutes in Europe
Source: Food Sci Nutr. 2025 Jan 12;13(1):e4753. doi: 10.1002/fsn3.4753 (PMC11725167; doi:10.1002/fsn3.4753)
Supplement: Supplementary file 1 — Data S1. [file FSN3-13-e4753-s001.docx]

**Section 1:** Consumers’ perceived benefits of reducing meat consumption by income group.

| Motivation factor | France (n = 1,000) | | | | Germany (n = 2,000) | | | |
| --- | --- | --- | --- | --- | --- | --- | --- | --- |
|  | L | M | H | P | L | M | H | P |
| … is a good way to save money | 44% | 42% | 39% | 44% | 44%^a^ | 41%^ab^ | 36%^b^ | 39%^ab^ |
| … is better for the environment | 31% | 35% | 38% | 31% | 40%^a^ | 44%^ab^ | 46%^b^ | 45%^ab^ |
| … helps to reduce the risk of disease | 18% | 23% | 23% | 19% | 22%^ab^ | 20%^a^ | 25%^b^ | 22%^ab^ |
| … is ethically correct | 18% | 20% | 24% | 18% | 21% | 26% | 25% | 23% |
| … makes you feel good | 18% | 17% | 22% | 10% | 22%^a^ | 24%^a^ | 31%^b^ | 26%^ab^ |
| … helps to manage weight | 18% | 17% | 15% | 19% | 17% | 19% | 19% | 17% |
| … gives you more energy | 18%^a^ | 12%^b^ | 10%^b^ | 6%^b^ | 9%^a^ | 10%^ab^ | 16%^b^ | 10%^ab^ |

| Motivation factor | Italy (n = 1,000) | | | | Spain (n = 1,000) | | | |
| --- | --- | --- | --- | --- | --- | --- | --- | --- |
|  | L | M | H | P | L | M | H | P |
| … is a good way to save money | 38%^a^ | 34%^ab^ | 27%^b^ | 33%^ab^ | 36%^a^ | 26%^b^ | 29%^ab^ | 25%^ab^ |
| … is better for the environment | 38%^a^ | 43%^ab^ | 50%^b^ | 32%^ab^ | 28%^a^ | 30%^ab^ | 37%^b^ | 30%^ab^ |
| … helps to reduce the risk of disease | 32% | 37% | 40% | 32% | 30% | 33% | 28% | 24% |
| … is ethically correct | 20% | 25% | 26% | 22% | 19%^a^ | 21%^ab^ | 29%^b^ | 13%^ab^ |
| … makes you feel good | 26% | 32% | 28% | 28% | 22% | 25% | 24% | 14% |
| … helps to manage weight | 19% | 24% | 24% | 15% | 29% | 26% | 33% | 16% |
| … gives you more energy | 11% | 8% | 9% | 8% | 12% | 12% | 7% | 2% |

*Note*: L = Low income (less than €1,500); M = Moderate income (€1,500–2,999); H = High income (€3,000 or more); P = Prefer not to disclose / Don’t know. Small sub-sample: A base size of 75 to 100 is considered low and is represented as (–). Data points are not displayed for groups with fewer than 75 respondents, as the sample size is too low to provide meaningful insights. Proportion Representation: Percentages reported in the tables are calculated within each socioeconomic group and represent the distribution of responses within these groups, rather than the overall percentage of the total population answering the question. For superscript: a, b: Different letters in the same column indicate statistically significant differences (*p*<0.05); ab: Indicates no significant difference from both "a" and "b" values.

**Section 2:** Consumers’ perceived benefits of reducing meat consumption by generation group.

| Motivation factor | France (n = 1,000) | | | | | | | Germany (n = 2,000) | | | | | | |
| --- | --- | --- | --- | --- | --- | --- | --- | --- | --- | --- | --- | --- | --- | --- |
|  | Z | M | YM | OM | X | BB | SG | Z | M | YM | OM | X | BB | SG |
| … is a good way to save money | 35%^a^ | 37%^a^ | 38%^a^ | 37%^a^ | 42%^ab^ | 50%^b^ | – | 39%^ab^ | 38%^ab^ | 41%^ab^ | 35%^a^ | 39%^ab^ | 43%^b^ | – |
| … is better for the environment | 39%^a^ | 31%^ab^ | 36%^ab^ | 28%^b^ | 33%^ab^ | 35%^ab^ | – | 42%^a^ | 38%^a^ | 38%^a^ | 39%^a^ | 40%^a^ | 49%^b^ | – |
| … helps to reduce the risk of disease | 27% | 20% | 20% | 20% | 20% | 21% | – | 21% | 21% | 24% | 20% | 24% | 22% | – |
| … is ethically correct | 27%^a^ | 28%^a^ | 28%^a^ | 29%^a^ | 16%^b^ | 14%^b^ | – | 30%^a^ | 26%^ab^ | 28%^ab^ | 24%^ab^ | 21%^ab^ | 22%^b^ | – |
| … makes you feel good | 19%^ab^ | 21%^ab^ | 17%^ab^ | 25%^a^ | 15%^b^ | 17%^ab^ | – | 30%^a^ | 27%^ab^ | 31%^ab^ | 24%^ab^ | 23%^b^ | 23%^b^ | – |
| … helps to manage weight | 16% | 19% | 15% | 23% | 16% | 17% | – | 22%^a^ | 23%^ab^ | 28%^ab^ | 19%^ab^ | 15%^b^ | 16%^b^ | – |
| … gives you more energy | 21%^a^ | 23%^a^ | 22%^a^ | 24%^a^ | 8%^a^ | 5%^b^ | – | 18%^a^ | 17%^a^ | 17%^a^ | 16%^a^ | 9%^a^ | 6%^b^ | – |

| Motivation factor | Italy (n = 1,000) | | | | | | | Spain (n = 1,000) | | | | | | |
| --- | --- | --- | --- | --- | --- | --- | --- | --- | --- | --- | --- | --- | --- | --- |
|  | Z | M | YM | OM | X | BB | SG | Z | M | YM | OM | X | BB | SG |
| … is a good way to save money | 31% | 35% | 34% | 36% | 32% | 36% | – | 31% | 27% | 30% | 25% | 32% | 33% | – |
| … is better for the environment | 45%^a^ | 41%^ab^ | 45%^ab^ | 39%^ab^ | 34%^b^ | 45%^ab^ | – | 32% | 29% | 26% | 31% | 27% | 34% | – |
| … helps to reduce the risk of disease | 30%^ab^ | 31%^ab^ | 25%^a^ | 35%^ab^ | 37%^ab^ | 39%^b^ | – | 26%^ab^ | 25%^a^ | 23%^ab^ | 27%^ab^ | 34%^b^ | 33%^b^ | – |
| … is ethically correct | 16%^a^ | 22%^ab^ | 18%^ab^ | 25%^ab^ | 20%^ab^ | 32%^b^ | – | 31%^a^ | 22%^ab^ | 25%^ab^ | 21%^ab^ | 17%^b^ | 19%^b^ | – |
| … makes you feel good | 23% | 27% | 25% | 28% | 29% | 32% | – | 25%^ab^ | 24%^ab^ | 27%^a^ | 21%^ab^ | 18%^b^ | 25%^ab^ | – |
| … helps to manage weight | 25% | 23% | 26% | 21% | 20% | 19% | – | 32%^ab^ | 27%^ab^ | 33%^a^ | 22%^ab^ | 30%^ab^ | 23%^b^ | – |
| … gives you more energy | 15%^a^ | 13%^a^ | 12%^a^ | 14%^a^ | 9%^a^ | 4%^b^ | – | 15%^a^ | 12%^ab^ | 14%^a^ | 11%^ab^ | 9%^ab^ | 7%^b^ | – |

*Note*: Z = Generation Z (25 and under); M = Millennials (26-41); YM = Younger Millennials (26-32); OM = Older Millennials (33-41); X = Generation X (42-57); BB = Baby Boomers (58-76); SG = Swing Generation / World War II (77+). Small sub-sample: A base size of 75 to 100 is considered low and is represented as (–). Data points are not displayed for groups with fewer than 75 respondents, as the sample size is too low to provide meaningful insights. Proportion Representation: Percentages reported in the tables are calculated within each socioeconomic group and represent the distribution of responses within these groups, rather than the overall percentage of the total population answering the question. For superscript: a, b: Different letters in the same column indicate statistically significant differences (*p*<0.05); ab: Indicates no significant difference from both "a" and "b" values.

**Section 3:** Factors influencing consumers’ preferences for purchasing meat substitutes by income group.

| Motivation factor | France (n = 1,000) | | | | Germany (n = 2,000) | | | |
| --- | --- | --- | --- | --- | --- | --- | --- | --- |
|  | L | M | H | P | L | M | H | P |
| 1. Sourced and produced locally | 32% | 21% | – | – | 19% | 23% | 18% | – |
| 1. Same taste/texture as the meat | 33% | 23% | – | – | 35% | 29% | 30% | – |
| 1. All natural ingredients | 19%^a^ | 32%^b^ | – | – | 27% | 31% | 28% | – |
| 1. Brand specializes in meat-free products | 17% | 24% | – | – | 18% | 20% | 22% | – |
| 1. Nutritionally rich | 18% | 21% | – | – | 23%^ab^ | 18%^a^ | 28%^b^ | – |
| 1. High vegetable/pulse content | 25% | 20% | – | – | 20% | 22% | 28% | – |
| 1. Versatility | 15% | 16% | – | – | 25% | 23% | 25% | – |
| 1. Brand that also produces meat products | 13% | 17% | – | – | 13%^a^ | 27%^b^ | 25%^b^ | – |
| 1. Environmental impact | 12% | 20% | – | – | 13% | 18% | 20% | – |
| 1. None of these | 9% | 7% | – | – | 7% | 5% | 3% | – |

| Motivation factor | Italy (n = 1,000) | | | | Spain (n = 1,000) | | | |
| --- | --- | --- | --- | --- | --- | --- | --- | --- |
|  | L | M | H | P | L | M | H | P |
| 1. Sourced and produced locally | 30% | 37% | – | – | 21% | 23% | – | – |
| 1. Same taste/texture as the meat | 29% | 24% | – | – | 28% | 28% | – | – |
| 1. All natural ingredients | 27%^a^ | 39%^b^ | – | – | 35% | 43% | – | – |
| 1. Brand specializes in meat-free products | 17% | 26% | – | – | 21% | 24% | – | – |
| 1. Nutritionally rich | 27% | 23% | – | – | 31% | 38% | – | – |
| 1. High vegetable/pulse content | 34% | 27% | – | – | 24% | 28% | – | – |
| 1. Versatility | 20% | 15% | – | – | 24% | 18% | – | – |
| 1. Brand that also produces meat products | 12% | 10% | – | – | 24% | 15% | – | – |
| 1. Environmental impact | 14% | 22% | – | – | 14% | 13% | – | – |
| 1. None of these | 4% | 6% | – | – | 2% | 2% | – | – |

*Note*: L = Low income (less than €1,500); M = Moderate income (€1,500–2,999); H = High income (€3,000 or more); P = Prefer not to disclose / Don’t know. Small sub-sample: A base size of 75 to 100 is considered low and is represented as (–). Data points are not displayed for groups with fewer than 75 respondents, as the sample size is too low to provide meaningful insights. Proportion Representation: Percentages reported in the tables are calculated within each socioeconomic group and represent the distribution of responses within these groups, rather than the overall percentage of the total population answering the question. For superscript: a, b: Different letters in the same column indicate statistically significant differences (*p*<0.05); ab: Indicates no significant difference from both "a" and "b" values.
